# Informing Early Intervention: Preschool Predictors of Anxiety Disorders in Middle Childhood

**DOI:** 10.1371/journal.pone.0042359

**Published:** 2012-08-08

**Authors:** Jennifer L. Hudson, Helen F. Dodd

**Affiliations:** 1 Centre for Emotional Health, Department of Psychology, Macquarie University, Sydney, New South Wales, Australia; 2 School of Psychology, University of East Anglia, Norwich, United Kingdom; University of Ottawa, Canada

## Abstract

**Background:**

To inform early intervention practice, the present research examines how child anxiety, behavioural inhibition, maternal overinvolvement, maternal negativity, mother-child attachment and maternal anxiety, as assessed at age four, predict anxiety at age nine.

**Method:**

202 children (102 behaviourally inhibited and 100 behaviourally uninhibited) aged 3–4 years were initially recruited and the predictors outlined above were assessed. Diagnostic assessments, using the Anxiety Disorders Interview Schedule, were then conducted five years later.

**Results:**

Behavioural inhibition, maternal anxiety, and maternal overinvolvement were significant predictors of clinical anxiety, even after controlling for baseline anxiety (p<.05). No significant effect of negativity or attachment security was found over and above baseline anxiety (p>.1).

**Conclusions:**

Preschool children who show anxiety, are inhibited, have overinvolved mothers and mothers with anxiety disorders are at increased risk for anxiety in middle childhood. These factors can be used to identify suitable participants for early intervention and can be targeted within intervention programs.

## Introduction

It is increasingly recognised that preschool children can experience clinically significant anxiety, with research reporting a prevalence rate of 9.5% for a community sample of children aged between 2 and 5 years. [1] A number of early intervention programs have recently been developed and evaluated and there is some evidence they are efficacious. To date, however, it is unclear which children should be prioritised for early intervention and what specifically should be targeted within an early intervention program. There is significant variation in program content and target population across trials. For example, the “Cool Little Kids” program teaches anxiety management skills to parents of children classified as behaviourally inhibited (BI). [2] In contrast, the “Being Brave” program combines psychoeducation for parents with child CBT and is designed for children with clinical anxiety disorders. [3] If the aim of early intervention is to decrease the presence of long-term anxiety disorders, then it is important to identify which factors lead to increased anxiety over time and, therefore, which variables should be targeted in treatment. With a view to informing practice in this way, the present research aims to identify the factors at age four that predict anxiety at age nine. Five variables were identified for evaluation as predictors: behavioural inhibition (BI; a temperament characterised by reactions of withdrawal, wariness, avoidance and shyness in novel, unfamiliar situations [4]); maternal psychopathology; maternal overinvolvement; maternal negativity; mother-child attachment. A number of factors influenced the selection of these variables. First, theoretical models of child anxiety and recent reviews of the literature suggest that these variables are associated with child anxiety over time. [5], [6] Second, as the aim of the research is to inform early intervention, it is vital that the variables examined as risk factors are amenable to change. Third, existing early intervention programs typically include modules that target mothers’ parenting and maternal anxiety so examination of their predictive utility is vital.

A link between BI in young children and subsequent anxiety is well-established in the literature. [7] However, not all inhibited children go on to experience clinically significant anxiety. It is currently unclear what additional factors affect anxiety risk in these children. Alongside research examining BI and anxiety is extensive research examining other risk factors for anxiety disorders. As outlined above, in this paper we are specifically interested in factors related to the family that have been theoretically and empirically linked to anxiety disorders and that might be amenable to change. For example, it is clear that children who have anxious parents are at risk for anxiety disorders themselves and parents. [8] In addition, there is some longitudinal evidence linking mother-child attachment to anxiety. [9] Furthermore, a large body of research has examined the association between parenting styles and anxiety in children. [10] Two parenting styles have primarily been examined: negativity/rejection and overinvolvement/control. Both overinvolvement and negativity have been associated with child anxiety, although findings are more consistent for overinvolvement. [11]

Research has only recently begun to examine BI and family environment factors together. It is possible that the family environment factors listed above act as additive risk factors, increasing anxiety risk in all children. Alternatively, as outlined in a number of theoretical models, family environment might interact with BI to affect anxiety risk. [12] To date a number of studies have been conducted examining temperament and family environment as predictors of anxiety over time but these have focused on older children, [13], [14] relied entirely on questionnaire measures, [15]–[17] or examined broader family process variables. [18], [19] This paper is unique in bringing together some of the dominant risk factors for child anxiety, based on previous research, and examining how these factors predict change in anxiety across a early childhood. It was hypothesised that each of the following, as assessed at age four, would be associated with child anxiety at age nine: 1) BI; 2) Maternal anxiety; 3) Maternal Overinvolvement; 4) Maternal negativity; 5) Attachment security. The extent to which each of these family environment factors predicted anxiety over and above concurrent anxiety at age four was evaluated by controlling for anxiety at baseline. In addition, following theoretical models that predict these family environment factors might moderate the relationship between BI and anxiety, interactions between BI and each of the four family environment factors listed above were examined. By examining a range of family environment predictors together with BI in a single study, using a multi-method design that includes diagnostic assessments at baseline, this research provides unique insights into preschool predictors of anxiety in middle childhood.

## Methods

This study presents a 5-year follow-up of a sample of behaviourally Inhibited (BI) and behaviourally uninhibited (BUI) children and their mothers. A detailed description of the sample, measures and results of the baseline assessments can be found in our earlier paper. [20]

### Participants

102 BI and 100 BUI children were initially recruited and baseline assessments were conducted when children were approximately age 4 (mean age: 48.2 months, sd = 4.26; 50% male). Of these, 71 BI and 89 BUI took part in the 5-year follow-up, when they were approximately age 9 (mean age: 106.74 months, sd = 3.61). Mean time between assessments was 58 months (sd = 2). Participants were initially recruited through local preschools and advertisements. BI classification was made at baseline on the basis of mothers’ report using the Short Temperament Scale for Children (STSC). [21] Children scoring 1 standard deviation above or below the normative mean on the Approach Scale were classified as BI or BUI respectively. There were an equal number of boys and girls in both temperament groups, 60% of the children were first born and the majority had one or more siblings (85%). Of the sample, 89% came from two-parent homes, 56% were from middle to high income families. Mothers were aged between 20 and 50 years (mean = 36.28 years, sd = 4.47 years). The majority of mothers (50%) stayed at home by choice, 42% worked part-time; 92% of mothers had completed school up to the age of 18 and 85% had obtained a post-school qualification. For ethnicity, 64% of participants were identified as being Oceanic, 20% as European and 10% as Asian, with the remainder being American, African or Middle Eastern. There were no significant differences between temperament groups for child age, maternal age, education, marital status, family income, number of siblings or birth order (p>.05). Significant differences were found for ethnicity, χ^2^ (5) = 11.87, p = .04, with greater numbers of children of Asian ethnicity in the BI group.

### Baseline Measures

#### Maternal-report of BI

BI was assessed at baseline using the approach scale of STSC, a parent-report measure containing 30 items. There are seven items that make up the Approach Scale. Example items are ‘My child is shy when first meeting new children’ and ‘When my family goes on a trip, my child immediately makes him/herself at home in the new surroundings’ (reverse-coded). High scores on the approach scale indicate lack of approach whereas low scores indicate approach behaviour. The STSC has adequate validity, good internal consistency and reliability. [21] The internal consistency for the approach scale in the present sample at baseline was α = .92.

#### Observed BI

BI was assessed at baseline using observed laboratory tasks developed in collaboration with Kagan and colleagues. [4] This protocol has been used in previous research conducted by Rapee and colleagues. [2] Children’s responses to a new room, novel toy, masked experimenter dressed in a strange suit and a same-sex unfamiliar peer were observed and coded in accordance with this previous research. Behaviours used to determine inhibition status included: i) time spent proximal to the mother; ii) amount of time spent staring at the peer; iii) time spent talking; iv) number of approaches to the stranger; and v) number of approaches to the peer. Participants were defined as BI based on observation if they scored above a pre-determined cut-off on ≥3 of these five behaviours. The cutoffs were: total time spent talking during stranger and peer components combined - less than 1 min; total time within arm’s length of mother during stranger and peer components combined - greater than 1 min; total time spent staring at peer - greater than 2 min; frequency of approach to stranger - one or less; frequency of approach to peer - one or less. Coding was conducted by postgraduate students in psychology, trained by the first author, who were blind to participants’ STSC scores and diagnoses. A second coder independently scored the videotapes for 25% of the sample. The inter-rater reliability for number of cutoffs exceeded was ICC = .91, and for overall BI classification was kappa = .79.

#### Child anxiety

Child anxiety diagnoses were assessed at baseline and 5-year follow-up using the Anxiety Disorders Interview Schedule for DSM-IV, parent/child version (ADIS-P-IV). [22] At baseline, only the mother was interviewed. At 5-year follow-up both the mother and child were interviewed and composite diagnoses were assigned. Interviews were conducted and diagnoses were assigned by trained psychologists who were unaware of the child’s group membership. Diagnoses were only considered ‘clinical’ if the clinical severity rating was 4 or greater. Twenty percent of the interviews were coded by a second clinician. Interrater agreement was as follows: presence of clinical anxiety diagnosis (baseline kappa = .86, 5-year follow-up kappa = .85), number of anxiety diagnoses (baseline ICC = .90, 5-year follow-up ICC = .90).

#### Maternal anxiety disorders

At baseline, mothers were interviewed using the Anxiety Disorders Interview Schedule for DSM-IV [23] to assess current and lifetime diagnoses. Diagnoses were assigned by trained clinicians unaware of the child’s group and anxiety status. To capture anxiety severity as well as clinical status, number of clinical anxiety diagnoses was used. A total of 20 cases (10%) were coded by a second clinician. Interrater agreement was as follows: number of current anxiety diagnoses (ICC = .85), number of lifetime anxiety diagnoses (ICC = .91).

#### Overinvolvement and Negativity

Maternal overinvolvement and negativity were assessed at baseline using a speech preparation task and the Five Minute Speech Sample (FMSS). Additionally, overinvolvement was assessed using the Parent Protection Scale (PPS). Each of these measures is described briefly below. [20] After converting the data from these measures to z-scores, means were calculated to construct a single overinvolvement variable and a single negativity variable.

The Parent Protection Scale (PPS) was used to assess maternal behaviours related to overprotection and autonomy granting. [24] The PPS contains 25 items (on a scale 0–3) and four subscales: Supervision, Separation, Dependence and Control. The Control scale was of interest to the current study and includes items such as ‘I determine who my child will play with’ and ‘I dress my child even if he/she can do it alone’. The PPS has adequate internal reliability, re-test reliability, criterion and content validity. [24], [25] The internal consistency in this sample was α = .65.

In the observation task, mothers were observed interacting with their child during a three-minute speech preparation task adapted from Hudson and Rapee. [26] The tasks were videotaped and maternal involvement and maternal negativity were coded by two trained individuals. Both coders were unaware of participants’ diagnostic status and rated each parent–child interaction. The reliability for the average of these ratings was ICC = .94 for the overinvolvement factor and ICC = .73 for the negativity factor.

The Five Minute Speech Sample (FMSS) was conducted and coded according to the method described by Magana and colleagues. [27] Mothers were asked to talk about their child and their relationship uninterrupted for 5 minutes. The speech samples were videotaped, transcribed and coded for criticism and over-involvement as outlined in the coding manual. [27] Coders were psychology students trained by the first author to the standard required by Magana et al. (1986). 24% of transcripts were assessed for inter-rater reliability: Overinvolvement (kappa = .63), Criticism (kappa = .96).

#### Attachment

At baseline, child-mother attachment was assessed using the preschool version of the Strange-Situation procedure. [28] Children were classified as having secure, insecure-avoidant, insecure-ambivalent, disorganised-controlling or insecure-other attachment following coding of videotaped interactions by one of two certified coders trained in the Cassidy-Marvin (Macarthur) Preschool Attachment Classification System. For the purposes of analyses, children were categorised into secure versus not secure. Twenty-one percent of cases were second-coded and reliability was kappa = .74.

### Procedures

Macquarie University Human Ethics Committee approved the procedures of the study including the consent procedures (reference: HE29NOV2002-R02087; HE30MAY2008-R05911). Mothers provided written consent for their and their child’s participation. Children provided verbal consent after being provided an explanation of the research. Written consent was not offered given the level of maturity of the children but children were included in discussions about consent and were given the opportunity to decline participation. Following the initial screen using the STSC, children meeting entry criteria were invited to take part in the full study and mothers provided written informed consent. At baseline and follow-up, participants visited the university for 2-hour sessions. In the follow-up assessments, child anxiety diagnoses were assessed and the questionnaire measures were completed.

### Statistical Analysis

Complete diagnostic data were available for 160 participants at 5-year follow-up. There were no significant differences between those who participated at 5-year follow-up and those that did not on child gender, maternal anxiety, maternal education, marital status, family income, ethnicity, or maternal age (*p*>.05). Participants who did not participate were, however, more likely to have been classified as BI and to meet criteria for an anxiety disorder at baseline. These data can be considered missing at random because data are missing as a function of an observed covariate. [29]

Both observation of temperament and parent-report of temperament have their limitations and discrepancy between parent-report and observation is often found in the temperament literature, with correlations typically around *r = *.3 to *r = *.4 ([30]). Based on behaviour during the laboratory assessment of BI, 92 participants were classified as inhibited and 110 participants as uninhibited. Classifications were in agreement with the original parent-report groups for 74% of participants. This is a relatively high rate of consistency, indicating that the observation of BI was externally valid. Given that the observation of BI is based on a short time-period and a limited range of circumstances, we chose to conduct our analyses primarily using the parent-report groups. Given the time and methodological constraints of conducting observation, parent-report measures are also much more practical when considering targeted intervention. All analyses were conducted again using the subsample of participants whose parent-report classification was consistent with their laboratory-based classification. This enabled us to be sure that the findings were not the result of bias in parent-report. Where differences in significance were found, these are reported.

A multi-method approach was taken for the analyses. The direct relationship between each independent variable (BI group; maternal number of current anxiety diagnoses; maternal number of lifetime anxiety diagnoses (includes current and past); maternal overinvolvement; maternal negativity; attachment security) and anxiety at 5-year follow-up was examined initially (dependent variables: presence of an anxiety diagnosis; number of anxiety diagnoses). As is common with count variables, number of child anxiety diagnoses conformed to a negative binomial (NB) distribution. Consequently NB regression was used when this was the dependent variable. For presence of an anxiety diagnosis, logistic regression was used. To assess whether each risk factor predicted anxiety at follow-up, over and above concurrent anxiety at baseline, the regressions were repeated for each IV, controlling for baseline anxiety. Finally, to examine whether the relationship between BI and anxiety was moderated by any of the family environment variables (maternal anxiety, maternal overinvolvement, maternal negativity, mother-child attachment) and to examine whether the family environment variables predicted anxiety at follow-up after controlling for BI as well as baseline anxiety, the regressions were conducted again. This time the regressions also included BI as an independent variable and an interaction term for each family environment variable and baseline BI group. Interaction terms were calculated by multiplying mean-centred variables. For all models that included BI, a dummy variable for Asian ethnicity was included.

## Results

Table 1 shows the prevalence rates for anxiety diagnoses at baseline and 5-year follow-up for the parent-report (PR) BI and BUI groups as well as significant between group differences at both timepoints.

**Table 1:** Prevalence rates of anxiety disorders at 5-year follow-up for parent-reported behaviourally inhibited (BI) and uninhibited (BUI) groups.

	Baseline^a^		5-year follow-up	
	BI	BUI	BI	BUI
Any anxiety disorder	68%	18%*	54%	18%*
Social Phobia	42%	0%*	37%	3%*
Separation Anxiety Disorder	34%	2%*	10%	1%*
Specific Phobia	45%	12%*	21%	11%
Generalised Anxiety Disorder	8%	3%	21%	9%*
Obsessive Compulsive Disorder	1%	2%	0%	0%
Post Traumatic Stress Disorder	1%	0%	0%	0%

a Baseline data are shown only for the N = 160 participants who completed 5-year follow-up.

* group differences are significant at time-point (*p*<.05).

### Predicting Presence of an Anxiety Diagnosis

The logistic regression analyses were conducted using SPSS version 18. Multiple imputation [31], [32] was utilised to create 20 data sets with complete follow-up data. All baseline variables were included as independent variables in the multiple imputation. The results reported below are for the pooled outcomes across these imputed datasets.

Table 2 shows the results for the logistic regressions examining the relationship between each risk factor and the presence of an anxiety diagnosis at 5-year follow-up. BI, maternal current and lifetime diagnoses and maternal overinvolvement predicted the presence of an anxiety diagnosis at follow-up, even after controlling for baseline anxiety. Maternal negativity and attachment did not predict anxiety at follow-up, regardless of whether baseline anxiety was controlled for. Baseline anxiety was a significant predictor in all analyses (*p*<.05).

**Table 2:** Results of logistic regressions to assess the effect of each risk factor on the presence of an anxiety diagnosis at 5-year follow-up.

Risk factor	Before controlling forbaseline anxiety	After controlling forbaseline anxiety	After controlling for baseline anxiety and BI group (PR)
Behavioural Inhibition (PR)	*b* = 1.48, *SE* = .38, *p*<.001, *OR* = 4.40	*b* = 1.10, *SE* = .42, *p* = .01, *OR* = 2.93	_
Number of maternal current anxiety disorders	*b* = .61, *SE* = .18, *p* = .001, *OR* = 1.84	*b* = .48, *SE* = .19, *p* = .01, *OR* = 1.62	*b* = .51, *SE* = .2, *p* = .01, *OR* = 1.7
Number of maternal lifetime anxiety disorders	*b* = .38, *SE* = .12, *p* = .002, *OR* = 1.47	*b* = .31, *SE* = .13, *p* = .02, *OR* = 1.36	*b* = .34, *SE* = .14, *p* = .01, *OR* = 1.4
Overinvolvement	*b* = .72, *SE* = .25, *p* = .004, *OR* = 2.05	*b* = .60, *SE* = .26, *p* = .02, *OR* = 1.82	*b* = 0.49, *SE* = .28, *p* = .08, *OR* = 1.63
Negativity	*b* = .36, *SE* = .24, *p* = .14, *OR* = 1.43	*b* = .18, *SE* = .26, *p* = .48, *OR* = 1.20	*b* = .1, *SE* = .28, *p* = .71, *OR* = 1.11
Attachment security	*b* = −.55, *SE* = .36, *p* = .12, *OR* = .58	*b* = −.42, *SE* = .38, *p* = .27, *OR* = .66	*b* = −.34, *SE* = .4, *p* = .4, *OR* = 0.72

Note: OR = Odds ratio.

To examine whether the BI-anxiety relationship was moderated by any of the family environment variables and to assess whether each family environment variable predicted anxiety at follow-up after controlling for BI as well as baseline anxiety, the above logistic regressions were repeated, this time including BI group and the interaction between BI group. None of the interaction terms were significant (*p*>.1). The interaction terms were therefore removed from the models. Table 2 shows the results for each family environment factor after controlling for group and baseline anxiety. Maternal current and lifetime diagnoses predicted the presence of an anxiety diagnosis at follow-up. The effect of overinvolvement approached significance.

When the analyses were conducted using only the data for participants with consistent BI classifications across parent-report and observation, the pattern of results was identical, except that the effect of anxiety at baseline was not a significant predictor when included in a model with BI (*b = *.483, *SE = *.48, *p = *.309, *OR = *1.62).

### Predicting Number of Anxiety Diagnoses

To handle the missing data for the negative binomial regressions, the Maximum Likelihood Ratio (MLR) algorithm and Monte Carlo Integration in MPlus Version 6 was used. [33] Table 3 shows the results of these analyses. The results are highly consistent with those reported above and indicate that parent report of BI, maternal current and lifetime diagnoses and maternal overinvolvement predicted number of anxiety diagnoses at follow-up, even after controlling for baseline anxiety (see Table 3). Prior to controlling for baseline anxiety, a significant effect of maternal negativity was found. This was not a significant predictor once baseline anxiety was controlled for (p>.05). Attachment security was not a significant predictor of anxiety at follow-up, even before baseline anxiety was controlled for. Baseline anxiety was a significant predictor in all analyses (p<.01).

**Table 3:** Results of negative binomial regressions to assess the effect of each risk factor on the number of child anxiety diagnoses at 5-year follow-up.

Risk factor	Before controlling forbaseline anxiety	After controlling forbaseline anxiety	After controlling for baseline anxiety and BI group (PR)
Behavioural Inhibition (PR)	*b* = 1.40, *SE* = .31, *p*<.001,IRR = 4.06	*b* = .97, *SE* = .37, *p* = .008, IRR = 2.64	_
Number of maternal current anxiety disorders	*b* = .47, *SE* = 0.08, *p*<.001, IRR = 1.60	*b* = .33, *SE* = .10, *p* = .001, IRR = 1.39	*b* = .32, *SE* = .01, *p* = .001, IRR = 1.38
Number of maternal lifetime anxiety disorders	*b* = .34, *SE* = .07, *p*<.001, IRR = 1.40	*b* = .24, *SE* = .1, *p* = .001, IRR = 1.27	*b* = .25, *SE* = .08, *p* = .001, IRR = 1.3
Overinvolvement	*b* = .63, *SE* = .19, *p* = .001, IRR = 1.88	*b* = .43, *SE* = .20, *p* = .03, IRR = 1.54	*b* = .34, *SE* = .019, *p* = .07, IRR = 1.41
Negativity	*b* = .38, *SE* = .19, *p* = .05, IRR = 1.46	*b* = 0.17, *SE* = .19, *p* = .39, IRR = 1.19	*b* = .05, *SE* = .18, *p* = .80, IRR = 1.01
Attachment security	*b* = −.51, *SE* = .28, *p* = .074, IRR = 0.60	*b* = −.32, *SE* = .27, *p* = .24, IRR = 0.73	*b* = .−18, *SE* = .25, *p* = .47, IRR = 0.83

Note: IRR = Incident Rate Ratio.

To examine whether the BI-anxiety relationship was moderated by any of the family environment variables and to assess whether each family environment variable predicted anxiety at follow-up after controlling for BI as well as baseline anxiety, the above NB regressions were repeated, this time including BI group and the interaction between BI group. None of the interaction terms were significant (*p*>.1). The interaction terms were therefore removed from the models. Table 3 shows the results for each family environment factor after controlling for group and baseline anxiety. Maternal current and lifetime diagnoses predicted the presence of an anxiety diagnosis at follow-up. The effect of overinvolvement approached significance.

When these analyses were conducted using the reduced sample of only participants with consistent BI classifications across parent-report and observation, the pattern of significance was identical, except that maternal negativity was not a significant predictor, even before baseline anxiety was controlled for (b = .11, SE = .21, p = .59, IRR = 1.12) and baseline anxiety was not a significant predictor when included in the same model as BI group (b = .21, SE = .12, p = .07, IRR = 1.23).

## Discussion

Although significant progress has been made in recent years in relation to the identification and treatment of anxiety in preschool children, it remains unclear which children should be targeted for early intervention and what the focus of early intervention should be. With this in mind, the aim of the present research was to identify factors that, at age four, predict anxiety in middle childhood. Five potential predictors of anxiety in middle childhood were selected based on the empirical literature and practical considerations with regards treatment content. Overall, the findings showed that preschool children are more likely to have a clinical anxiety diagnosis in middle childhood when they: show early anxiety; are behaviourally inhibited; have mothers who are more overinvolved; have mothers with anxiety disorders.

Whilst extensive research has demonstrated that BI is associated with increased risk for anxiety, the present study addresses the important question of whether BI contributes to the prediction of anxiety over and above concurrent anxiety by assessing both BI and anxiety at baseline. Even at age four, a high proportion of the sample met criteria for an anxiety diagnosis. These rates are relatively high, but are consistent with other research with similar populations. [34] The results provide clear support for BI as a predictor of child anxiety over time; BI at age 4 was associated with increased risk for social phobia, separation anxiety disorder and generalised anxiety disorder at age 9. Furthermore, BI remained a significant predictor of anxiety at age 9, even when the significant effect of anxiety at age 4 was controlled for. This indicates that, at least to some extent, BI and anxiety represent independent constructs that both affect risk for anxiety over time. Interestingly, when parent-report and observation of BI were combined, BI was actually a stronger predictor of anxiety over time than baseline anxiety. Of additional interest here is the change in risk over time for specific anxiety diagnoses. Although there was a consistent risk for BI children at age 4 and 9 for social phobia, the risk for specific phobia was only present at age 4. In contrast, a new risk emerged for BI children at age 9: BI children were more likely than BUI children to have a diagnosis of GAD. This pattern of decreasing specific phobia and increasing GAD over time is consistent with epidemiology of anxiety disorders.

The results provide clear support for a role of maternal anxiety in affecting child anxiety; both current and lifetime maternal anxiety were strong predictors of child anxiety. In addition, there was some evidence that maternal parenting was also predictive of anxiety. It was hypothesised that maternal overinvolvement and maternal negativity would predict child anxiety over time. The results for overinvolvement supported this hypothesis; overinvolvement was a significant predictor of child anxiety at age 9, even when baseline anxiety was controlled for. In contrast, there was little evidence that maternal negativity affected child anxiety over time. This pattern of findings is in keeping with the results of a meta-analysis showing larger effect sizes for overinvolved or intrusive parenting than negative parenting. [11] The final aspect of the mother-child relationship that was assessed was attachment security. The findings gave no indication that attachment security was predictive of anxiety at follow-up. These findings do not suggest that attachment is not important for other aspects of child development. It is also important to consider that attachment was assessed at age 4; so it remains possible that attachment in earlier life could be a predictor of anxiety in middle childhood.

One of the strengths of the present research is that multiple family environment factors were assessed alongside behavioural inhibition, including maternal anxiety, maternal parenting and mother-child attachment. It has been proposed that these family environment factors might moderate the relationship between BI and anxiety over time. [5], [6] In order to accurately capture which children are most likely to go on to be clinically anxious in middle childhood, it is important that these interactions are also considered. The potential moderating effect of each of the family environment variables studied was examined but none of the interactions were found to be significant. This suggests that the variables examined confer additive risk for anxiety disorders. Although this finding is not consistent with theoretical models, which hypothesise temperament by environment interactions, [13] it is in keeping with earlier research examining the prediction of anxiety symptoms. For example, of 16 possible temperament by parenting interactions assessed, Kiff and colleagues found only three that were significant in predicting change in anxiety: two for the temperament effortful control and one for irritability. No significant interactions were found between fearful temperament (related to BI) and parenting. [13] Where significant interactions have been found in previous research, these have been in the prediction of internalising problems in general, [35] rather than for child anxiety. [15], [36]

There are a number of reasons that these anticipated interactions were not observed. As Kiff et al. discuss, it is possible that temperament by environment interactions differ according to the child’s gender. [12] We do not have the power to assess this hypothesis in the present sample but this is an important question for future research. It is also possible that the difficulty in finding these interactions consistently is an artefact of the difficulty finding interactions in non-experimental research. [37] Finally, it is possible that children high on BI are not any more vulnerable to adverse environments than those low on BI and that these risk factors simply have additive effects on child anxiety. It is important to keep in mind here that these family environment factors might be particularly important for BI children because they increase the child’s already high-risk status. The increased risk conferred by overinvolved parenting may be inconsequential to a child who is temperamentally low risk for anxiety.

### Implications for Early Intervention

The findings have clear implications for early intervention. Child anxiety, BI, maternal anxiety and maternal overinvolvement as assessed at age four were all significant predictors of child anxiety at age nine. These factors can therefore be used to identify children who are at risk for long-term anxiety problems and can provide some focus for the content of early intervention programs. To address inhibition and anxiety in preschool children, intervention programs could incorporate exposure hierarchies and modules on recognising anxiety, generating brave thoughts and coping skills. The findings highlight the important role that mothers play in affecting child anxiety, via their own anxiety and their parenting. It is therefore essential that mothers are active participants in intervention. In relation to overinvolvement, modules on reducing overprotection and increasing the child’s independence are likely to be useful. Given the importance of maternal anxiety as a predictor, decreasing the mother’s anxiety through exposure and cognitive restructuring is also recommended. The efficacy of interventions that specifically target these factors needs to be evaluated.

### Strengths and Limitations

The present study has a number of strengths: 1) a thorough methodology was used incorporating questionnaire measures, behavioural observation and diagnostic interviews; 2) child anxiety and BI were assessed at baseline, which allowed their respective value as predictors to be examined; 3) participants were followed from the point at which early intervention is usually conducted into middle childhood and a good retention rate was achieved; 4) several family environment factors were assessed alongside BI, which allowed for moderation effects to be examined. In relation to limitations, first, only maternal factors were examined. There has been a call in recent years for increased attention to fathers’ roles in the development of anxiety in children with recent research showing that maternal and paternal factors have independent affects on children’s anxiety. [38], [39] Future research examining the relative importance of mothers and fathers will provide important insights into whether it is of value to include both parents in early intervention programs. A second point that requires consideration is that all of the variables that predict anxiety in middle childhood include maternal report to some extent. It is possible therefore, that the relationship between these variables and diagnoses at follow-up is the result of shared method variance. This seems unlikely for several reasons. First, these variables predict anxiety after controlling for baseline anxiety; any shared method variance would also be captured in the baseline diagnostic assessment and therefore controlled for when this variable was included. Second, the diagnoses at follow-up were made using a well-validated clinical diagnostic instrument and both mothers and children were interviewed by an experienced interviewer, the extent to which mothers own biases might affect this outcome is therefore minimised. Finally, the nature of the sample should be taken into consideration. The sample was self-selected and participants were largely from two-parent, middle to high income homes. It will therefore be important to attempt to replicate these findings in other samples, including a sample who have a lower socio-economic background.

A further limitation is the sample size; although a relatively large sample was recruited, the sample was not large enough to examine all of the predictors and all possible interactions in a single model. Also, due to the age of the children in the baseline assessment, we were unable to obtain child report. The inclusion of child report at the follow-up may have lead to more cases being detected. Finally, it is important to acknowledge that only five potential risk factors for child anxiety were included in the present study. Other risk factors may also play an important role in the development and maintenance of anxiety. For example, Degnan and colleagues [6] discuss the role that peers and caregiving outside of the immediate family might play in child anxiety. Areas of interest for future research include more detailed evaluation of early intervention programs with specific reference to which children benefit the most, which components are particularly useful, whether individual or group interventions are more effective and whether children’s attendance significantly improves outcome as compared to parent-only interventions

## References

[1] Egger HL, Angold A (2006) Common emotional and behavioral disorders in preschool children: presentation, nosology, and epidemiology. J Child Psychol Psychiat 47: 313–337. 16492262 10.1111/j.1469-7610.2006.01618.x

[2] Rapee RM, Kennedy S, Ingram M, Edwards S, Sweeney L (2005) Prevention and Early Intervention of Anxiety Disorders in Inhibited Preschool Children. J Consult Clinic Psychol 73: 488–497. 10.1037/0022-006X.73.3.488 15982146

[3] Hirshfeld-Becker D, Masek B, Henin A, Blakely L, Pollock-Wurman R, et al (2010) Cognitive behavioral therapy for 4- to 7-year-old children with anxiety disorders: a randomized clinical trial. J Consult Clinic Psychol 78: 498–510. 10.1037/a0019055 20658807

[4] Garcia-Coll C, Kagan J, Reznick J (1984) Behavioral Inhibition in young children. Child Dev 55: 1005–1019.

[5] Hudson JL, Rapee RM (2004) From Anxious Temperament to Disorder: An Etiological Model of Gerneralized Anxiety Disorder. In: Heimberg RG, Turk CL, Mennin, DS editors Generalized anxiety disorder: Advances in research and practice. (. xvi, 2446 p. New York, NY: Guilford Press. 2051–2074.

[6] Degnan KA, Almas AN, Fox NA (2010) Temperament and the environment in the etiology of childhood anxiety. J Child Psychol Psychiatry 51: 497–517. 20158575 10.1111/j.1469-7610.2010.02228.x PMC2884043

[7] Chronis-Tuscano A, Degnan KA, Pine DS, Perez-Edgar K, Henderson HA, et al (2009) Stable Early Maternal Report of Behavioral Inhibition Predicts Lifetime Social Anxiety Disorder in Adolescence. J Am Acad Child Psychiatry 48: 928–935 910.1097/CHI.1090b1013e3181ae1009df. 10.1097/CHI.0b013e3181ae09df PMC2789287 19625982

[8] Cooper PJ, Fearn V, Willetts L, Seabrook H, Parkinson M (2006) Affective disorder in the parents of a clinic sample of children with anxiety disorders. J Affect Disord 93: 205–212. 16675030 10.1016/j.jad.2006.03.017

[9] Moss E, Smolla N, Cyr C, Dubois-Comtois K, Mazzarello T, et al (2006) Attachment and behavior problems in middle childhood as reported by adult and child informants. Dev Psychopathol 8: 425–444. 10.1017/S0954579406060238 16600062

[10] McLeod BD, Wood JJ, Avny SB (2011) Parenting and Child Anxiety Disorders. In: McKay D, Storch EA, editors. Handbook of Child and Adolescent Anxiety Disorders. New York: Springer. 213–228.

[11] McLeod BD, Wood JJ, Weisz JR (2007) Examining the association between parenting and childhood anxiety: A meta-analysis. Clin Psychol Rev 27: 155–172. 17112647 10.1016/j.cpr.2006.09.002

[12] Kiff C, Lengua L, Zalewski M (2011) Nature and Nurturing: Parenting in the Context of Child Temperament. Clin Child Fam Psychol Rev 14: 251–301. 21461681 10.1007/s10567-011-0093-4 PMC3163750

[13] Kiff CJ, Lengua LJ, Bush NR (2011) Temperament variation in sensitivity to parenting: predicting changes in depression and anxiety. J Abnorm Child Psychol 39: 1199–1212. 21800017 10.1007/s10802-011-9539-x PMC3199341

[14] Schwartz O, Dudgeon P, Sheeber L, Yap M, Simmons J, et al (2012) Parental Behaviors During Family Interactions Predict Changes in Depression and Anxiety Symptoms During Adolescence. J Abnorm Child Psychol 40: 59–71. 21789522 10.1007/s10802-011-9542-2

[15] Muris P, van Brakel AML, Arntz A, Schouten E (2011) Behavioral inhibition as a risk factor for the development of childhood anxiety disorders: A longitudinal study. J Child Fam Stud 20: 157–170. 21475710 10.1007/s10826-010-9365-8 PMC3048305

[16] Edwards SL, Rapee RM, Kennedy S (2010) Prediction of anxiety symptoms in preschool-aged children: Examination of maternal and paternal perspectives. J Child Psychol Psychiatry 51: 313–321. 19769584 10.1111/j.1469-7610.2009.02160.x

[17] Hudson JL, Dodd HF, Bovopoulous N Temperament and family environment in the development of anxiety disorder: Two-year follow-up. J Am Acad Child Psychiatry. In press. 10.1016/j.jaac.2011.09.009 22115146

[18] Mian N, Wainwright L, Briggs-Gowan M, Carter A (2011) An Ecological Risk Model for Early Childhood Anxiety: The Importance of Early Child Symptoms and Temperament. J Abnorm Child Psychol 39: 501–512. 21153696 10.1007/s10802-010-9476-0 PMC5179257

[19] Volbrecht MM, Goldsmith HH (2010) Early temperamental and family predictors of shyness and anxiety. Dev Psychol 46: 1192–1205. 20822232 10.1037/a0020616 PMC3086562

[20] Hudson JL, Dodd HF, Bovopoulos N Temperament, Family Environment and Anxiety in Preschool Children. J J Abnorm Child Psychol 39. In press. 10.1007/s10802-011-9502-x 21479669

[21] Sanson A, Prior M, Garino E, Oberklaid F, Sewell J (1987) The structure of infant temperament: Factor analysis of the Revised Infant Temperament Questionnaire. Infant Behav Dev 10: 97–104.

[22] Silverman WK, Nelles WB (1988) The Anxiety Disorders Interview Schedule for Children. J Am Acad Child Psychiatry 27: 772–778. 10.1097/00004583-198811000-00019 3198566

[23] DiNardo PA, Barlow DH (1990) Syndrome and symptom co-occurrence in the anxiety disorders. Maser JD, Cloninger CR, editors. Comorbidity of mood and anxiety disorders (pp 205–230) xvii, 869pp.

[24] Thomasgard M, Metz W, Edelbrock C, Shonkoff JP (1995) Parent-child relationship disorders: I. Parental overprotection and the development of the Parent Protection Scale. Dev Behav Pediatr 16: 244–250. 7593659

[25] Thomasgard M, Metz W (1999) Parent-child relationship disorders: What do the child vulnerability scale and the parent protection scale measure? Clin Pediatr 38: 347–356. 10.1177/000992289903800605 10378092

[26] Hudson JL, Rapee RM (2001) Parent–child interactions and anxiety disorders: An observational study. Behav Res Ther 39: 1411–1427. 11758699 10.1016/s0005-7967(00)00107-8

[27] Magana AB, Goldstein MJ, Karno M, Miklowitz DJ, Jenkins J, et al (1986) A brief method for assessing expressed emotion in relatives of psychiatric patients. Psychiatry Res 17: 203–212. 3704028 10.1016/0165-1781(86)90049-1

[28] Cassidy J, Marvin RS (1992) Group wtMW (1992) Attachment organization in preschool children: Procedures and coding manual. University of Virginia: Unpulished manuscript.

[29] Schafer JL, Graham JW (2002) Missing data: Our view of the state of the art. Psychol Methods 7: 147–177. 12090408

[30] Kochanska G, Clark LA, Goldman MS (1997) Implications of mothers’ personality for their parenting and their young children’s developmental outcomes. J Pers 65: 387–420. 9226943 10.1111/j.1467-6494.1997.tb00959.x

[31] Rubin DB (1987) Multiple imputation for nonresponse in surveys. New York: Wiley.

[32] Sinharay S, Stern HS, Russell D (2001) The use of multiple imputation for the analysis of missing data. Psychol Methods 6: 317–329. 11778675

[33] Muthén LK, Muthén BO (1998–2010) Mplus User’s Guide. 6th ed. Los Angeles, CA: Muthén & Muthén..

[34] Rapee RM, Kennedy SJ, Ingram M, Edwards SL, Sweeney L (2010) Altering the Trajectory of Anxiety in At-Risk Young Children. Am J Psychiatry. appi.ajp.2010.09111619. 10.1176/appi.ajp.2010.09111619 20810472

[35] Rankin-Williams L, Degnan KA, Perez-Edgar KE, Henderson HA, Rubin KH, et al (2009) Impact of behavioral inhibition and parenting style on internalizing and externalizing problems from early childhood through adolescence. J Abnorm Child Psychol 37: 1063–1075. 19521761 10.1007/s10802-009-9331-3 PMC2791524

[36] Shamir-Essakow G, Ungerer JA, Rapee RM (2005) Attachment, Behavioral Inhibition, and Anxiety in Preschool Children. J Abnorm Child Psychol 33: 131–143. 15839492 10.1007/s10802-005-1822-2

[37] McClelland GH, Judd CM (1993) Statistical difficulties of detecting interactions and moderator effects. Psychol Bull 114: 376–390. 8416037 10.1037/0033-2909.114.2.376

[38] Bogels S, Phares V (2008) Fathers’ role in the etiology, prevention and treatment of child anxiety: A review and new model. Clin Psychol Rev 28: 539–558. 17854963 10.1016/j.cpr.2007.07.011

[39] Pahl KM, Barrett PM, Gullo MJ (2012) Examining potential risk factors for anxiety in early childhood. J Anxiety Disord 26: 311–320. 22261038 10.1016/j.janxdis.2011.12.013

